# Tension Hydrothorax from Disseminated Endometriosis

**DOI:** 10.5811/westjem.2015.11.28503

**Published:** 2016-01-12

**Authors:** AnnaKate Deal, David Evans, Francis L. Counselman

**Affiliations:** * Eastern Virginia Medical School, Department of Emergency Medicine, Norfolk, Virginia; † Virgina Commonwealth University, Department of Emergency Medicine, Richmond, Virginia; ‡ Emergency Physicians of Tidewater, Norfolk, Virginia

## INTRODUCTION

We present the case of a 34-year-old woman presenting to the emergency department (ED) with dyspnea, cough, and fever. She was found to have a tension hydrothorax and was treated with ultrasound-guided thoracentesis in the ED. Subsequent inpatient evaluation showed the patient had disseminated endometriosis. Tension hydrothorax has not been previously described in the literature as a complication of this disease.

## CASE REPORT

A 34-year-old Nigerian woman presented to the ED with a chief complaint of dyspnea, cough, and fever for two days. The patient also complained of epigastric pain and intermittent emesis. She denied any significant prior medical or surgical history.

Physical exam revealed a toxic-appearing female in respiratory distress. Vital signs were temperature 100.5°F; blood pressure 103/76mmHg; heart rate 126bpm; respiratory rate 32bpm; and 99% oxygen saturation on room air. She had decreased breath sounds on the right side, and exhibited right upper quadrant tenderness on palpation. The cardiovascular exam was remarkable only for the tachycardia.

**Figure 1:**
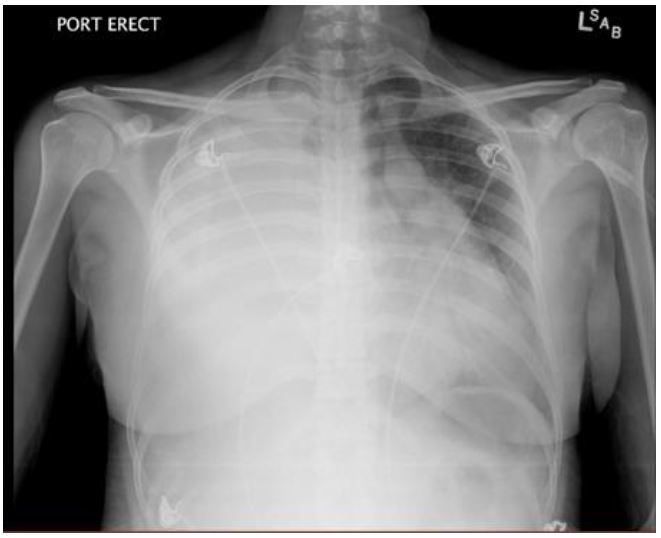
Portable anteroposterior radiograph of the chest reveals complete atelectasis of the right lung (white arrow), adjacent right-sided pleural effusion (arrowhead) and leftward mediastinal shift (black arrow) consistent with tension hydrothorax.

**Figure 2:**
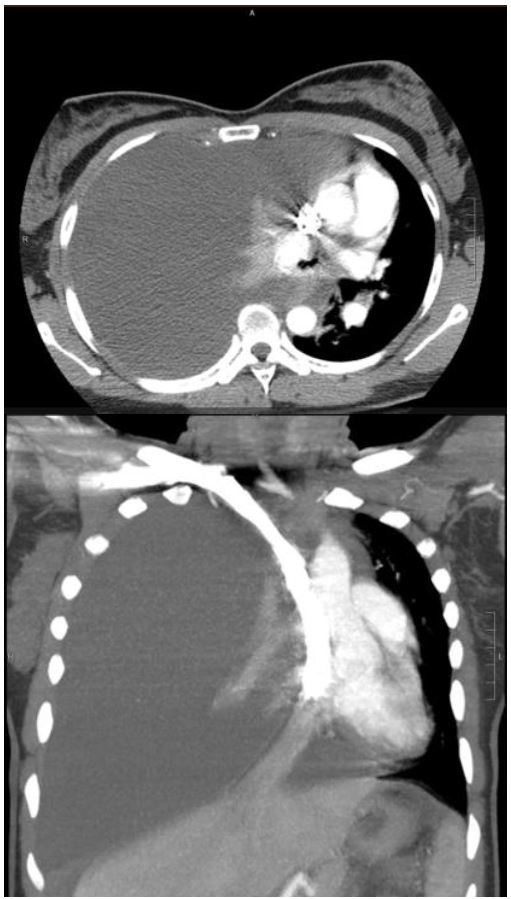
Axial (A) and coronal (B) computed tomography angiogram images of the chest showing a large right pleural effusion (arrows) and near-complete atelectasis of the right lung (arrow heads) with leftward mediastinal shift.

A portable chest radiograph (CXR) revealed complete atelectasis of the right lung with adjacent large right pleural effusion, causing mediastinal shift to the left, consistent with tension hydrothorax (Figure 1). A computed tomography (CT) angiogram of the chest confirmed the above findings; there was no evidence of pulmonary embolism or aortic dissection (Figure 2). An ultrasound-guided thoracentesis removed 1.5L of serosanguinous fluid. The patient reported relief of chest pain and dyspnea after the thoracentesis.

The patient was admitted to the hospital and treated for presumed pneumonia and sepsis with broad spectrum antibiotics. A purified protein derivative for tuberculosis was negative. She had a chest tube placed on hospital day 2 for recurrent right pleural effusion, with return of two liters of serosanguinous fluid. Pleural fluid cultures, acid fast bacteria (AFB) and cytology were all negative.

Because of worsening abdominal pain and distention, on hospital day 4 a CT of the abdomen/pelvis was ordered and revealed a pelvic mass of unclear uterine or adnexal etiology, with abdominal implants and ascites, suspicious for malignancy. A follow–up pelvic ultrasound was less suggestive of malignancy, and revealed two solid uterine lesions and an enlarged right ovary with cyst. Magnetic resonance imaging of the abdomen and pelvis revealed bilateral complex cystic adnexal structures with hemorrhage; a tubo-ovarian abscess was suspected.

On hospital day 14, the patient underwent laparoscopy with biopsy. Laparoscopy revealed blue-tinged lesions on the central omentum, a nodular omental lesion, and pelvic wall nodule. The peritoneum was described as having a fibrinous exudate with mucinous appearance along the surface. Biopsies of the omental and pelvic nodules confirmed the lesions to be consistent with endometriosis and a pelvic wall abscess. The final diagnosis was disseminated endometriosis resulting in tension hydrothorax and pelvic inflammatory disease. The patient was discharged on hospital day 22 to complete an outpatient course of antibiotics.

## DISCUSSION

Tension hydrothorax is defined as a large pleural effusion that increases intrathoracic pressure enough to cause a shift in mediastinal structures and decrease venous return.^1^ It differs from tension pneumothorax in that symptoms may develop slowly over time until a critical intrathoracic pressure is reached. As fluid accumulates in the pleural space, this causes the lung volume on the affected side to decrease, resulting in tachypnea, decreased breath sounds, and hypoxia.^2^ As the volume and pressure within the pleural cavity increases, venous return to the right ventricle decreases.^2^ If untreated, tension hydrothorax and tension pneumothorax share a common terminal pathophysiology. Tachycardia, hypoxemia, jugular venous distention, and hypotension are observed due to the severe elevation of intrathoracic pressure in conjunction with decreased venous return and compression of the right ventricle.^2^

In the adult population, malignancy and infection are the primary causes of tension hydrothorax.^1^ Additional causes described in the literature include: ventriculopleural shunting;^2^,^3^ peritoneal dialysis;^4^,^5^ migration of ventriculoperitoneal shunts;^1^ cirrhosis (hepatic hydrothorax);^6^ ovarian hyperstimulation syndrome;^7^ and central venous catheterization.^8^

Most patients will present with obvious pulmonary pathology, including dyspnea, decreased or absent breath sounds, tachypnea and hypoxia. A CXR will confirm the diagnosis. Depending upon the patient’s acuity, a chest CT can provide additional information. Findings on laboratory studies are inconsistent, and depend on the etiology of the tension hydrothorax. Management of tension hydrothorax involves immediate drainage. Depending on the patient’s hemodynamics, this may involve therapeutic thoracentesis, or more commonly, tube thoracostomy. This typically will result in immediate improved hemodynamics, improved oxygenation and decreased dyspnea. Once drained, the pleural fluid should be sent for gram stain, cultures (aerobic and anaerobic), AFB and cytology. Additional studies may be required to identify the etiology of the pleural fluid and allow for definitive management.

Interestingly, we could not find a case of tension hydrothorax related to endometriosis in the literature. The thorax is a rare site of endometriosis.^7^ There are two forms of thoracic endometriosis: the pulmonary and the pleural form. The pulmonary form presents as catamenial hemoptysis and pulmonary nodules. The pleural form includes catamenial pneumothorax (pneumothorax associated with menstruation); catamenial hemothorax; catamenial pneumomediastinum; and chest pain.^7^ Of these, catamenial pneumothorax is the most common manifestation of thoracic endometriosis (73%), followed by catamential hemothorax (14%), catamenial hemoptysis (7%) and lung nodules (6%).^7^ The pathogenesis of thoracic endometriosis is not well understood; the two predominant theories are microembolization and peritoneal-pleural based migration.^7^ Interestingly, the right hemothorax is involved in more than 90% of all cases of thoracic endometriosis,^7^,^9^ and the peak incidence of thoracic endometriosis is between 30 and 34 years old;^9^ our patient clearly fit the profile.

## CONCLUSION

We describe the first published case of a tension hydrothorax presenting as a complication of disseminated endometriosis in a young woman.
